# Influence of Three Combinations of Cryoprotectants and Two Warming Temperatures on Cellular Morphology, Morphometry and Mitochondrial Activity of Vitrified Canine Testicles

**DOI:** 10.1111/rda.70074

**Published:** 2025-05-14

**Authors:** Juliana de Souza Fernandes, Jéssyka Araújo Noronha, Francisco Denilson Rodrigues Gomes, Bruna Farias Brito, Gisele Karla Sena Guimarães, Herlon Victor Rodrigues Silva, Leda Maria Costa Pereira Bersano, Lúcia Daniel Machado da Silva

**Affiliations:** ^1^ Laboratório de Reprodução de Carnívoros (LRC) Universidade Estadual do Ceará (UECE) Fortaleza CE Brazil; ^2^ Laboratório de Manipulação de Oócitos e Folículos Ovarianos Pré‐Antrais (LAMOFOPA) Universidade Estadual do Ceará (UECE) Fortaleza CE Brazil; ^3^ Centro Universitário Fametro (UNIFAMETRO) Fortaleza CE Brazil

**Keywords:** cryopreservation, dimethyl sulfoxide, ethylene glycol, glycerol, male gonad

## Abstract

High concentrations of cryoprotectants required for testicular vitrification result in a toxic environment for cells. To mitigate this issue, a suitable alternative is to combine cryoprotectants. The temperature for warming a vitrified sample is also important to assure cell viability. Thus, the aim of this work was to evaluate how combining cryoprotectants (ethylene glycol‐EG, glycerol‐GLY, and dimethyl sulfoxide‐DMSO) in pairs and using two warming temperatures (37°C and 50°C) influence cellular morphology, tubular morphometry, and mitochondrial activity after testicular vitrification of dogs. Testicular fragments from ten adult dogs were distributed among the fresh control group (CTR) and the experimental groups according to the combination of cryoprotectants and temperatures (EG/GLY37, EG/GLY50, DMSO/GLY37, DMSO/GLY50, DMSO/EG37 and DMSO/EG50). The fragments were vitrified in a final concentration of 5.6 mol/L (2.8 mol/L of each of the cryoprotectants combined two by two) and subsequently warmed up to 37°C/30 s or 50°C/5 s. Following this, they were processed for histomorphological, morphometric, and mitochondrial activity evaluations with Rhodamine 123. In the morphometric evaluation, all vitrified groups showed a significant reduction in tubular diameter (*p* < 0.05). All experimental groups showed greater basement membrane separation when related to the CTR (*p* < 0.05). DMSO/EG37 showed the greatest basement membrane separation when compared to all other groups (*p* < 0.05). Regarding membrane retraction, all vitrified groups, regardless of the warming temperature, had greater retraction when related to CTR (*p* < 0.05), except DMSO/GLY50, which did not differ from any group (*p* > 0.05). Regarding the distinction between spermatogonia and Sertoli cells, no groups warmed up to 50°C differed from the control, except DMSO/GLY37. For nuclear visualisation, none of the vitrified groups differed from the CTR (*p* > 0.05), except DMSO/GLY37 (*p* < 0.05), which showed better nuclear visualisation. For the nuclear condensation parameter, there were no significant differences among the groups (*p* > 0.05). Mitochondrial activity was reduced in all vitrified samples, regardless of the combination of cryoprotectants and warming temperature (*p* > 0.05). It was concluded that the association of DMSO/GLY50 presented better preservation of morphological aspects.

## 1 Introduction

Testicular fragment cryopreservation techniques allow long‐term preservation of the reproductive potential of individuals. In addition, they promote the biodiversity of species, reducing the threat of extinction through the creation of germplasm banks (Lima et al. 2018a; Silva et al. 2012, 2020).

In this context, testicular vitrification presents itself as a promising possibility for preserving the reproductive potential of animals due to its reduced costs, quick execution, and good results (Silva 2022). Nonetheless, vitrification requires the use of high concentrations of cryoprotectants, which can cause damage to samples due to their toxicity (Lima et al. 2018a, 2018b). Therefore, the combination of cryoprotectants is used as a way to reduce their concentrations and, consequently, reduce the toxicity of these agents (Poels et al. 2012; Pukazhenthi et al. 2015).

Testicular vitrification with the combination of dimethyl sulfoxide (DMSO) and glycerol (GLY) has already been tested on testicular fragments from prepubertal cats. This combination presented the best results regarding the maintenance of cell morphology and viability when compared to the DMSO/ethylene glycol (EG) and EG/GLY combinations (Fernandes et al. 2021; Lima et al. 2016). In pubertal dogs, testicular vitrification with the DMSO/EG (ethylene glycol) combination stood out with better results compared to other cryoprotectant associations (Teixeira et al. 2021).

In order to assess the quality of testicular samples after the vitrification procedure, some parameters can be used. Among the parameters that can be used, we chose the morphometrical evaluation that included the measurement of the diameter of the seminiferous tubules; the morphological evaluation that included the evaluation of cell separation from the basement membrane, basement membrane retraction, distinction between spermatogonia and Sertoli, nuclear visualisation and nuclear condensation, and finally, the evaluation of mitochondrial activity that was assessed using Rhodamine 123. A positive finding would be considered if after testicular vitrification there was no modification of any of these parameters compared to fresh testicular samples (Fernandes et al. 2021).

After the vitrification process, the testicular fragment needs to be able to maintain active spermatogenesis, producing sperm that can later be used for in vitro fertilisation (Silva 2022). It is important to consider that the warming step after vitrification can be decisive, as it is capable of changing tissue/cellular integrity due to the risks of recrystallisation, toxic factors, oxidative, and osmotic stress that samples may suffer (Lima et al. 2018a, 2018b). The warming process is essential to improve the endurance of vitrified samples and optimise cryopreservation and tissue warming protocols (Volkova et al. 2021). In studies in which temperatures of 37°C and 50°C were compared for postvitrification testicular warming, the highest temperature better preserved cell morphology and viability (Fernandes et al. 2021; Lima et al. 2016; Teixeira et al. 2021).

Some studies have already explored the effects of combining cryoprotectants in dogs (Teixeira et al. 2021) and other studies have addressed the effects of heating temperature on cat testicles (Fernandes et al. 2021; Lima et al. 2016, 2018a and 2018b). Still, it has not yet been possible to establish the ideal protocol for testicular vitrification in dogs. Therefore, the aim of this work was to evaluate the influence of three cryoprotectant combinations (EG/GLY, DMSO/GLY, DMSO/EG) and two warming temperatures (37°C and 50°C) on cellular morphology, tubular morphometry, and mitochondrial activity after testicular vitrification in dogs.

## 2 Materials and Methods

### 2.1 Ethical Aspects

The Animal Ethics Committee of the State University of Ceará (UECE) approved this work under protocol number 109556951/2019.

### 2.2 Animals

In this study, 10 testicular pairs from 10 healthy adult male dogs, aged between 1 and 5 years, were evaluated. The canines underwent elective bilateral orchiectomy at Jacó Veterinary Clinic (VetMóvel unit) in the city of Fortaleza, Ceará.

### 2.3 Obtaining Testicular Fragments and Adding Cryoprotectants

The testicles obtained were immediately transported to the laboratory using a sterile 50 mL plastic tube containing 0.9% saline at room temperature (~22°C) within 1 h. Then, the testes were dissected with a scalpel blade in Ham's F‐10 medium (Sigma Aldrich, N6908). Twelve fragments from the testicular pair parenchyma measuring 3 × 3 × 1 mm were obtained, of which 3 fragments were randomly divided for each experimental group: fresh control (CTR), vitrified with the combination of ethylene glycol (Neon Company, ref. 00730) and glycerol (Neon Company, ref. 01456) (EG/GLY); dimethyl sulfoxide (Neon Company, ref. 03014) and glycerol (DMSO/GLY); dimethyl sulfoxide and ethylene glycol (DMSO/EG).

### 2.4 Testicular Vitrification

The testicular fragments were vitrified using the needle immersion method. For this, three fragments were transfixed in 30G (0.3 mm diameter × 13 mm length) hypodermic needles. Two fragments were intended for classical histology, and one was for fluorescence analysis. These fragments were initially immersed in an equilibrium solution with a total volume of 1.8 mL. The equilibrium solution was composed of 1.4 mol/L of each cryoprotectant from the three tested combinations (EG/GLY, DMSO/GLY, DMSO/EG), 0.25 mol/L of sucrose (Neon, ref. 02086), and minimal essential medium with Ham's F10 (Nutrient Mixture, Ham's F10), for 10 min at room temperature (22°C). Then, the fragments were subjected to vitrification solution (total volume: 1.8 mL), which contained 2.8 mol/L of each cryoprotectant (final concentration of 5.6 mol/L) in Ham's F10 medium supplemented with 10% foetal bovine serum (FBS) (Gibco Brazil, ref. 12657‐029 and 0.50 mol/L sucrose, for 5 min at room temperature (22°C) (Lima et al. 2016; Wang et al. 2008).

The hypodermic needles containing testicular fragments, after being submitted to the equilibration and vitrification solutions, were immediately immersed in liquid nitrogen for vitrification (Lima et al. 2016; Wang et al. 2008). Still immersed, the fragments were transferred to previously identified cryotubes, containing liquid nitrogen inside at liquid nitrogen temperature. Then, they were stored in a cryogenic cylinder for a minimum period of one week.

### 2.5 Warming of Testicular Fragments

**FIGURE 1:**
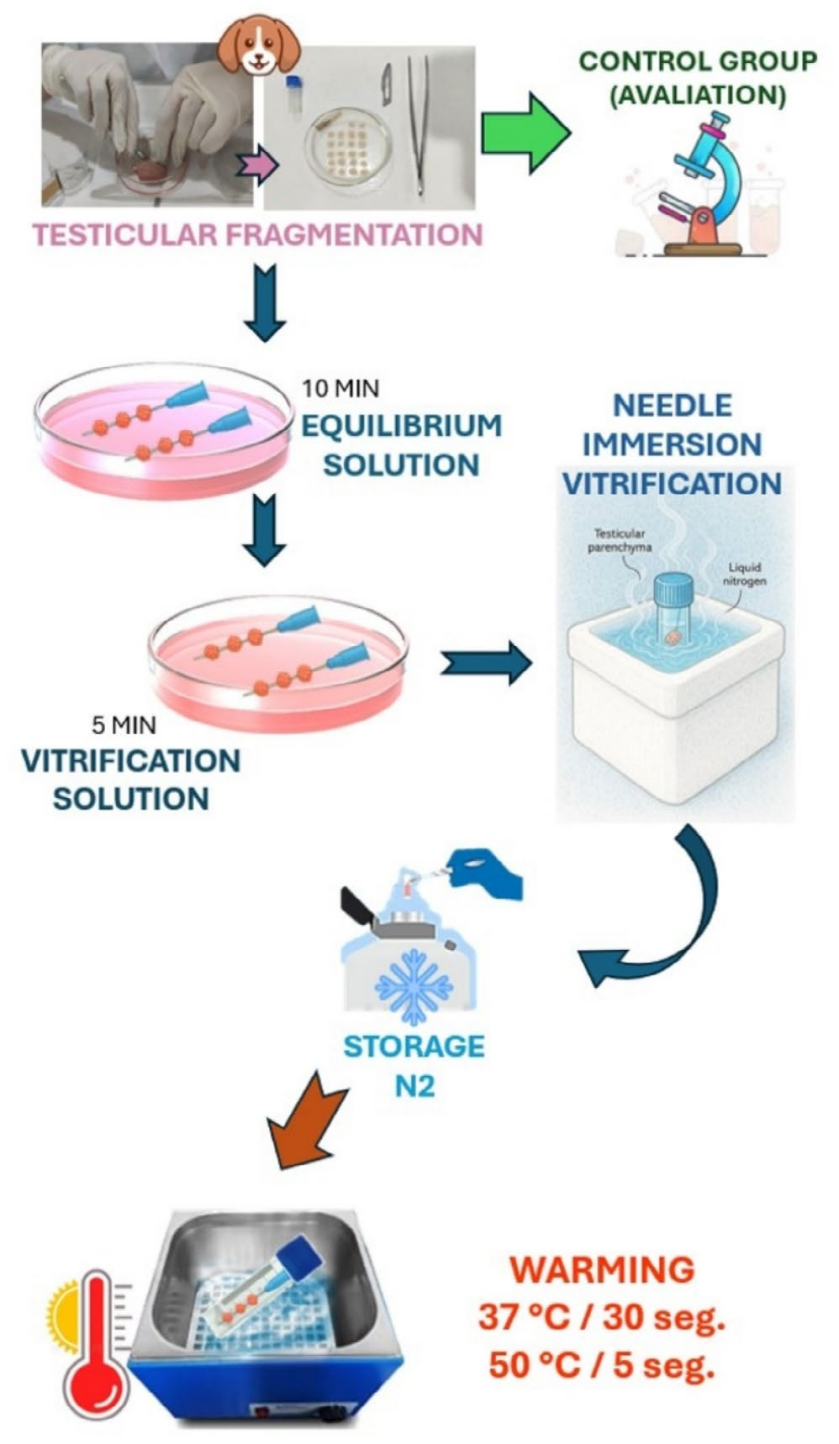
Experimental design illustrating the flow of the canine testicular vitrification and heating experiment.

After the storage period, the cryotubes were removed from the nitrogen cylinders and the needles containing testicular fragments were immersed in prewarmed PBS solution. In the groups warmed up to 37°C, referred to as EG/GLY37, DMSO/GLY37 and DMSO/EG37, the needles remained at room temperature for 1 min (~22°C) before being immersed for 30 s in PBS at 37°C. In the groups warmed up to 50°C, referred to as EG/GLY50, DMSO/GLY50 and DMSO/EG50, the needles containing the testicular fragments were removed from liquid nitrogen and promptly immersed for 5 s in PBS warmed up to 50°C (Fernandes et al. 2021). Then, the fragments from each experimental group were subjected to immersion baths in solutions of decreasing order of sucrose (0.50, 0.25 and 0 mol/L) added with Ham's F10 and 20% FBS. The baths were carried out at room temperature (~22°C) for 5 min to remove excess of cryoprotectants (Lima et al. 2018b) (Figure 1).

### 2.6 Histomorphometric and Histomorphological Evaluation

Testicular fragments of the CTR and those obtained after warming were fixed in Bouin's (Êxodo Científica Brazil, ref. FB08835SO) solution for 24 h at 4°C for histological processing. Then, they were taken to the histological routine following the steps of dehydration, diaphanisation, paraffin embedding, blocking, microtomy (5 μm), deparaffinisation, slide mounting, and staining with haematoxylin–eosin (Lima et al. 2018b).

The slides were evaluated under optical microscopy (Nikon Eclipse Ci‐L microscope; Nikon, Tokyo, Japan) with digital photomicrography (Nikon DS‐Fi3 camera, USB 3.0, 5.9 megapixel CMOS image sensor) at 200×.

A total of 20 seminiferous tubules from each sample were morphometrically evaluated regarding their diameter. Using NIS Elements software (Nikon, Nikon Instruments Inc.), this measure was calculated as the average of the two largest measurements from a basal membrane to one opposite of the same seminiferous tubule in cross section and perpendicular to each other), using the NIS Elements software (Silva et al. 2009; Teixeira et al. 2021).

For morphological analyses, thirty seminiferous tubules (ST) were randomly selected for each group and each animal, totalling 300 tubules (10 animals/group × 30 tubules). Then, they were evaluated according to five parameters: cell separation from the basement membrane, basement membrane retraction, spermatogonia/Sertoli distinction, nuclear visualisation, and nuclear condensation. For each parameter, a score that ranged from 1 to 3 was assigned, with 1 corresponding to normal morphology, 2 to discrete or moderate changes, and 3 to severe changes (Lima et al. 2016—Table 1).

**TABLE 1:** Histomorphological parameters of seminiferous tubules from canine testicles.

Parameters	Score 1	Score 2	Score 3
Cell separation from the basal membrane	None	Separation < 75%	Separation > 75%
Basal membrane retraction	None	Little retraction	Exacerbated retraction
Distinction spermatogonia/Sertoli	Clear	Not well defined	Impossible distinction
Nuclear visualisation	Easy	Difficult	Impossible
Nuclear condensation	None	< 50% of cells with nuclear condensation	> 50% of cells with nuclear condensation

*Note:* Adapted from Lima et al. (2016).

### 2.7 Assessment of Mitochondrial Activity

Rhodamine 123 solution (Invitrogen, Thermo Fisher Scientific, R302) was used to evaluate mitochondrial membrane activity using fluorescence microscopy (Santos et al. 2009). For this, testicular fragments from all groups were sliced with the aid of a scalpel blade so that they became smaller and smaller. Right away, they were incubated in a dark chamber in a 1.5 mL plastic tube containing 1 μL of a solution composed of 10 μg/mL of Rhodamine 123 in Ham's F10 medium supplemented with 10% FBS (Lima et al. 2018a). This medium was kept for 15 min in a water bath at 37°C. After incubation, three washes using PBS were performed, lasting 1 min each, at room temperature, to remove fluorochrome in excess. The washes were performed with drops of PBS, carried out in the same conical tube (Eppendorf) in which the incubation was performed. The drop of PBS from the wash was deposited on the fragment and gravity, and the inclination of the tube were able to make this drop descend to the bottom of the tube, leaving the fragment ready for the subsequent wash. Subsequently, the fragments were mounted on a slide (Olen Company, ref. K5‐7105‐1) and coverslip (Olen Company, ref. K5‐2450) with one drop (approximately 2 μL) of Prolong Gold Antifade reagent (Invitrogen—Thermo Fisher Scientific, ref. P36930). Images were obtained immediately after mounting the slides using an epifluorescence microscope (Nikon Eclipse Ci‐L microscope; Nikon, Tokyo, Japan) (Nikon light source, C‐HGFI) and measured with Nikon‐NIS Software Elements, with 40× magnification and blue filter (emission from 420 to 550 nm). Microscope settings and camera parameters were the same throughout the experiment.

The pixel intensity emitted by Rhodamine 123 was determined by NIS software (Nikon, Eclipse 80i, Tokyo, Japan) by analysing 10 randomly selected areas with the same dimension for all samples. The relative intensity was determined by subtracting the Rhodamine 123 fluorescence emission from the background (dark) of each slide (Fernandes et al. 2021) adapted from Lima et al. (2018b). For comparison purposes, the fluorescence emission of the control group was considered the normal standard. Fluorescence emission greater or less than the standard was considered high or low, respectively (Tanpradit et al. 2016).

### 2.8 Statistical Analysis

All data were analysed using the statistical software R‐project version 3.3.2 (The R Foundation, Vienna, Austria). In order to compare the means, the 95% confidence interval was considered. All results were expressed as mean ± standard deviation.

For morphological analyses, the data were submitted to the Cramer–Von Mises normality test and the Box–Cox homoscedasticity test. Parametric data such as basement membrane separation and nuclear condensation were submitted to ANOVA, followed by the Student–Newman–Keuls test. Nonparametric data, such as basement membrane retraction, spermatogonia/Sertoli distinction and nuclear visualisation, were submitted to the Kruskal–Wallis test, followed by Dunn multiple comparison.

For morphometry and mitochondrial membrane activity, data were submitted to the Cramer–Von Mises normality test and the Box–Cox homoscedasticity test. The data were parametric and analysed using ANOVA, followed by the Student–Newman–Keuls test.

## 3 Results

### 3.1 Histomorphometric Evaluation

**FIGURE 2:**
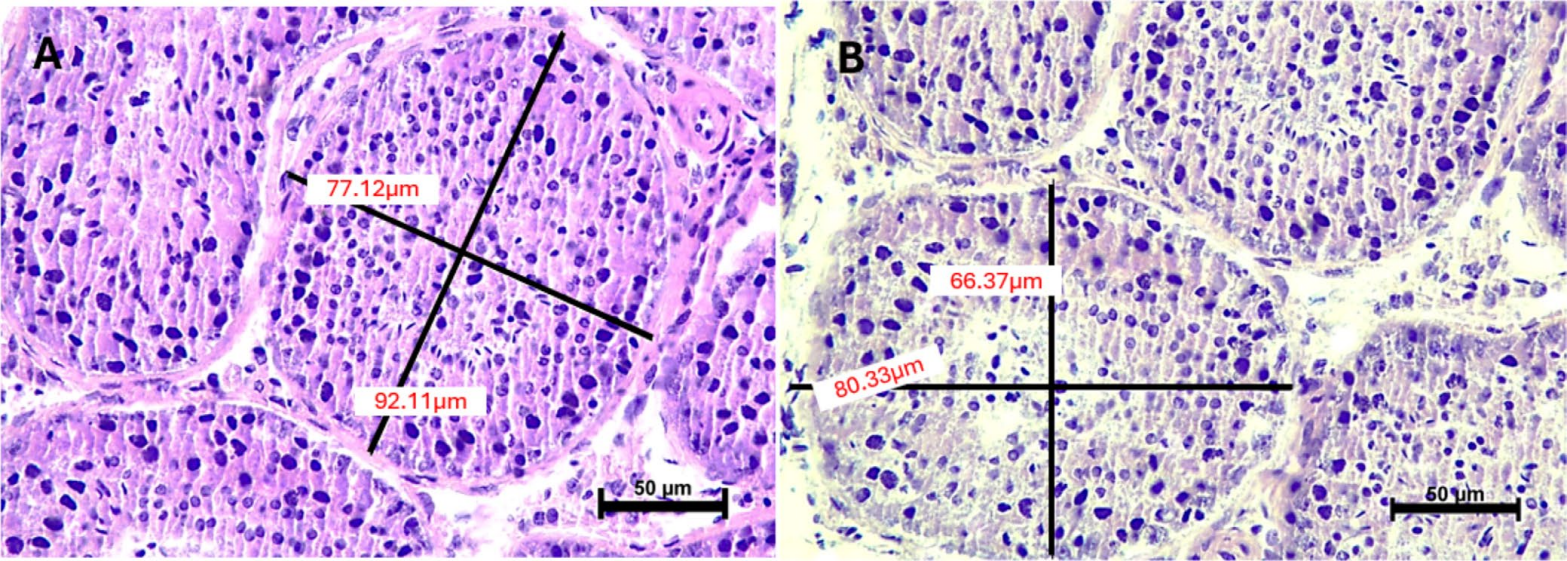
Morphometry of vitrified and warmed canine testicles in different protocols. HE staining, 400× magnification (scale 50 μm). (A) Control group (CTR). (B) Morphometry of the DMSO/GLY50 seminiferous tubule.

The tubular diameter, regardless of the warming temperature and the association of cryoprotectants, was significantly reduced (*p* < 0.05) in all vitrified groups when compared to the control group (Table 2, Figure 2).

**TABLE 2:** Morphometry (seminiferous tubule diameter—STD) of canine testicles vitrified under different cryoprotectant associations and warmed at 37°C or 50°C.

Parameters	Fresh	EG/GLYC37	EG/GLYC50	DMSO/GLY37	DMSO/GLY50	DMSO/EG37	DMSO/EG50
STD (μm)	88.01 ± 6.55^aA^	77.09 ± 7.25^bB^	72.99 ± 6.03^bB^	77.60 ± 5.89^bB^	79.43 ± 6.61^bB^	77.46 ± 7.47^bB^	75.14 ± 6.28^bB^

*Note:* ^a,b^Different lowercase letters indicate differences between extenders associations for the same warming temperature (*p* < 0.05). ^A,B^Different uppercase letters indicate differences between extenders associations for different warming temperatures (*p* < 0.05).

### 3.2 Histomorphological Evaluation

All experimental groups showed greater basement membrane separation when compared to the control (*p* < 0.05). DMSO/EG combination at the warming temperature of 37°C presented the greatest basement membrane separation when compared to all other groups (*p* < 0.05). Regarding membrane retraction, all vitrified groups, regardless of the warming temperature, had greater retraction in relation to the control (*p* < 0.05), except for the DMSO/GLY combination at the warming temperature of 50°C, which did not differ from any group (*p* > 0.05).

**FIGURE 3:**
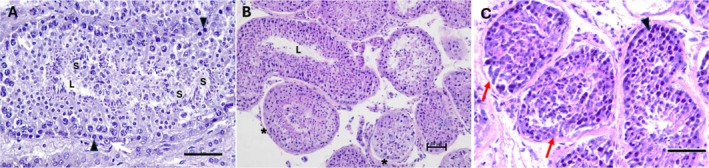
Morphology of vitrified and heated canine testis in different protocols. HE staining, 200× and 400× magnification (scale 50 μm). (A) Control group (CTR) demonstrating spermatogenesis. (B) and (C) Morphological changes in structures and cell populations after vitrification and warming (white arrow: Sertoli cell; arrowhead: spermatogonia; asterisk: separation and retraction of basement membrane; S: spermatozoa in the lumen of the tubule; L: lumen of the seminiferous tubule; red arrow: poor differentiation of spermatogonia and Sertoli cells).

Regarding the distinction between spermatogonia and Sertoli cells, all groups warmed to 50°C did not differ from the control, as did DMSO/GLY warmed to 37°C. For the nuclear visualisation parameter, none of the vitrified groups differed from the control (*p* > 0.05), except DMSO/GLY warmed to 37°C (*p* < 0.05), which showed better nuclear visualisation. For the nuclear condensation parameter, there were no significant differences among the groups (*p* > 0.05) (Table 3, Figure 3).

**TABLE 3:** Morphological analysis (Table 1 parameters) of canine testicles vitrified under different associations of cryoprotectants and warmed at 37°C or 50°C.

Parameters	Fresh	EG/GLY37	EG/GLY50	DMSO/GLY37	DMSO/GLY50	DMSO/EG37	DMSO/EG50
Cell separation BM	1.13 ± 0.11^a^	1.41 ± 0.11^b^	1.33 ± 0.11^b^	1.35 ± 0.12^b^	1.40 ± 0.12^b^	1.56 ± 0.20^c^	1.29 ± 0.13^b^
BM retraction	1.01 ± 0.02^a^	1.50 ± 0.18^b^	1.60 ± 0.12^b^	1.47 ± 0.11^b^	1.41 ± 0.10^ab^	1.46 ± 0.16^b^	1.44 ± 0.15^b^
Distinction s/Sertoli	1.10 ± 0.11^a^	1.33 ± 0.15^b^	1.09 ± 0.76^a^	1.18 ± 0.11^ab^	1.20 ± 0.08^a^	1.31 ± 0.16^b^	1.10 ± 0.06^a^
Nuclear visualisation	1.13 ± 0.12^a^	1.02 ± 0.03^ab^	1.04 ± 0.04^a^	1.01 ± 0.02^b^	1.03 ± 0.04^a^	1.09 ± 0.06^a^	1.06 ± 0.05^a^
Nuclear condensation	1.37 ± 0.34^a^	1.43 ± 0.14^a^	1.35 ± 0.14^a^	1.27 ± 0.17^a^	1.44 ± 0.14^a^	1.28 ± 0.07^a^	1.26 ± 0.08^a^

*Note:* ^a,b,c^Different lowercase letters on the same line indicate that there was a significant difference between groups (*p* < 0.05).

Abbreviations: BM retraction, basal membrane retraction; Cell separation BM, Cell separation from the basal membrane; Distinction s/Sertoli, distinction between spermatogonia and Sertoli cell.

### 3.3 Assessment of Mitochondrial Activity

**FIGURE 4:**
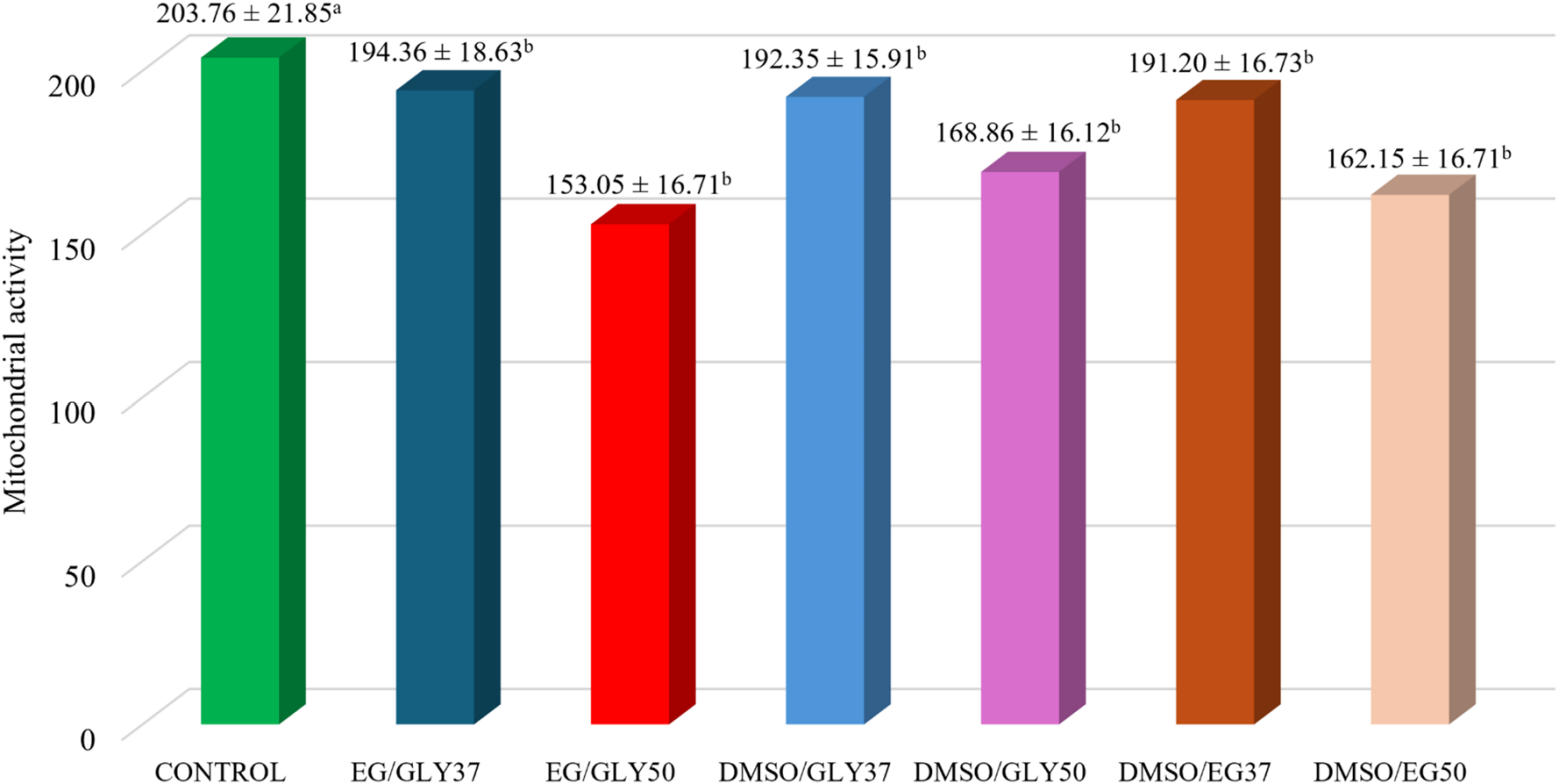
Evaluation of mitochondrial activity using Rhodamine 123 of canine testicular fragments. Fresh control group (CONTROL) and vitrified groups with different cryoprotectant combinations: EG/GLY, DMSO/GLY, DMSO/EG, and warmed at 37°C or 50°C. ^a,b^Different lowercase letters indicate that there was a significant difference between groups (*p* < 0.05).

**FIGURE 5:**
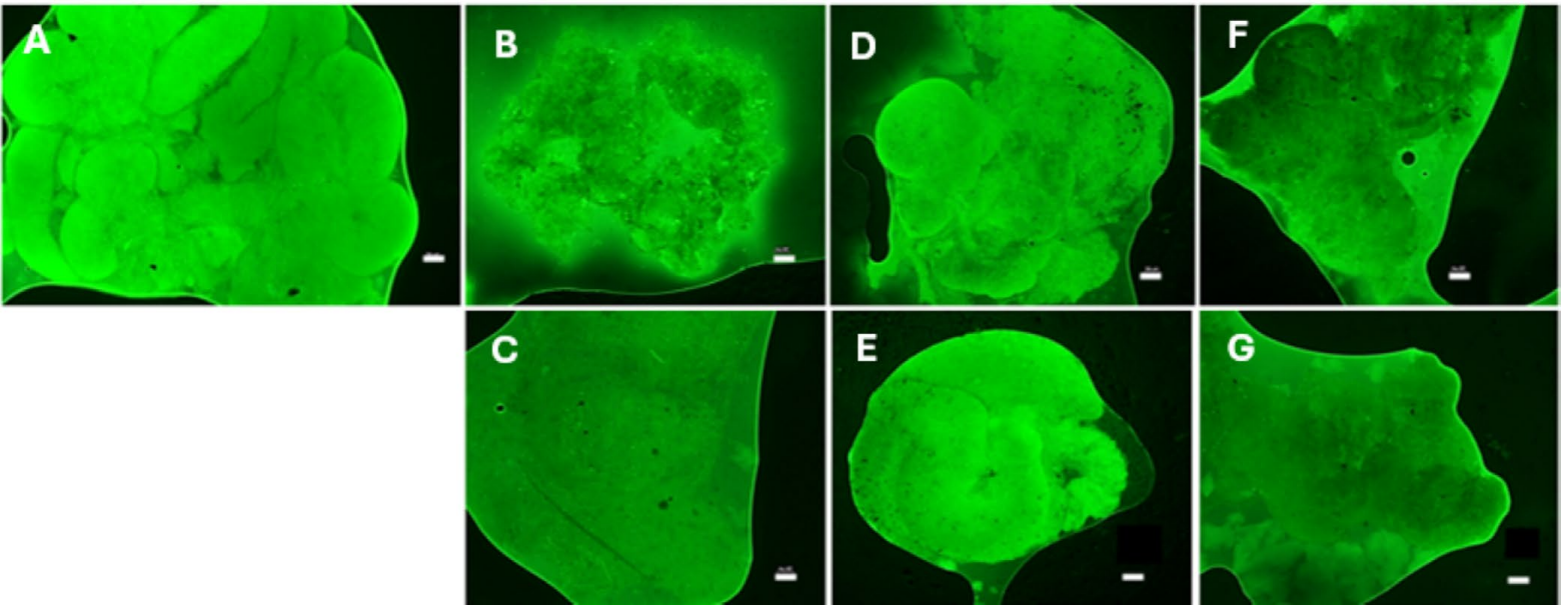
Testicular viability by mitochondrial activity with incidence of Rhodamine 123. (A) Control group (CTR); (B) EG/GLY37; (C) EG/GLY50; (D) DMS0/GLY37; (E) DMSO/GLY50. (F) EG/DMSO37. (G) EG/DMSO50 (measurement of fluorescence intensity in the testicular fragment). Magnification of 40× (scale of 50 μm).

Regarding mitochondrial activity assessed by fluorescence intensity, a similar behaviour was observed regarding the tubular diameter, in which there was a significant reduction in intensity (*p* < 0.05) in all vitrified groups when compared to the control group (Figures 4 and 5).

## 4 Discussion

Cryopreservation of testicular fragments represents a promising technique to preserve the potential of male gonads. Nevertheless, one of the challenges of this technique is to overcome the deleterious effects of the toxicity of cryoprotective agents (Comizzoli et al. 2012) and warming after cryopreservation, since both can affect cellular cryoresistance (Picazo et al. 2022). In order to mitigate these deleterious effects, three combinations of cryoprotectants were tested. The DMSO/GLY combination warmed up to 50°C was the one that best preserved morphological integrity after vitrification, like what was observed in studies on cats and dogs, respectively (Lima et al. 2018b; Teixeira et al. 2021). The EG/GLY and DMSO/EG groups, both also warmed up to 50°C, were like the control in three (distinction of spermatogonia and Sertoli cells, condensation and nuclear visualisation) out of the five parameters evaluated. The integrity of these three parameters demonstrates the efficiency of the protocol chosen for cell maintenance. Furthermore, since nuclear visualisation is an initial and basic characteristic to characterise cell preservation, the ideal would be for the observed characteristics to be similar to those of fresh samples. Therefore, warming up to 50°C promotes better maintenance of morphological characteristics compared to combinations of cryoprotectants in which warming was carried out at 37°C.

In studies in which vitrification was performed by needle immersion of testicular fragments from prepubertal cats, the authors observed that the DMSO/GLY combination with warming up to 50°C better preserved the morphological characteristics of the samples (Fernandes et al. 2021; Lima et al. 2018b). This present work also came to these results, although it was carried out with another species (dog) and another age group (adult). This fact denotes that the combination of DMSO/GLY cryoprotectants and the warming temperature of 50°C are both recommended for canine species in adulthood.

On the other hand, the DMSO/EG combination best preserved the viability and integrity of testicular DNA in dogs (Picazo et al. 2022) and grey wolves ( *Canis lupus* ) (Andrae et al. 2021). These results demonstrate the importance of establishing specific protocols that consider not only the species, but also the age of the animal, since the behaviour of testicular fragments of prepubertal dogs differed from those of adult animals when submitted to vitrification.

Satisfactory cell preservation has been reported after testicular cryopreservation using different combinations with DMSO, GLY and EG as cryoprotectants in dogs (Teixeira et al. 2021), mice (Gossens et al. 2008) and rats (Volkova et al. 2021), as well as in the maintenance of spermatogonial proliferation in nonhuman primates (Poels et al. 2012). DMSO has stood out as a cryoprotectant in testicular cryopreservation, whether it is alone or used in combination, providing satisfactory results (Silva 2022). DMSO has high solubility in water, which provides fast penetration. Thus, it is a better choice when compared to propanediol, due to its greater effectiveness in protecting the seminiferous tubules from cryogenic damage, resulting in the maintenance of testicular structures (Keros et al. 2005; Milazzo et al. 2008; Thuwanut et al. 2013) and cell viability. Glycerol, despite having low solubility in water, can provide cryogenic protection by increasing the viscosity of the extracellular medium and reducing the chance of formation of ice crystals (Barbosa et al. 2009). Therefore, the DMSO/GLY combination appears to provide the beneficial properties of each one of the cryoprotectants in the testicular vitrification of dogs.

The warming temperature of 50°C for testicular fragments resulted in better structural conservation and testicular mitochondrial activity when compared to the temperature of 37°C in cats (Fernandes et al. 2021; Lima et al. 2018a), rats (Volkova et al. 2021), dogs and wild boars (Picazo et al. 2022). This work also demonstrated that testicular warming to 50°C was better at preserving morphology. However, regarding mitochondrial activity, neither the different associations of cryoprotectants nor the warming temperature had a positive effect.

It is necessary to comprehend the importance of warming conditions, as well as establish the advantages and critical points of this process. Elucidating and establishing these characteristics are essential to improve the survival of testicular cells postvitrification. The warming temperature of 50°C is still considered an alternative to the temperature of 37°C. However, the hypothesis that vitrification is compatible with a proportionally fast warming, such as that carried out in the present work, has already been raised and strengthened (Lima et al. 2018b; Picazo et al. 2022; Volkova et al. 2021).

Testicular cryopreservation in dogs is still in its early stages of research. However, with the growing interest in cryobiology, it is expected that advances in this field will contribute not only to canine reproduction, but also to its application in wild canids. All cryopreservation techniques involve risks of damage to cellular structures, which can reduce cell viability. Although progress has been made, there is still no ideal standardisation for the process of testicular cryopreservation in dogs, which remains an open research field.

## 5 Conclusion

Using the combination of DMSO/GLY and warming at 50°C, testicular fragments from pubescent dogs submitted to vitrification by needle immersion showed better preservation of morphological aspects.

## Author Contributions

**Juliana de Souza Fernandes, Jéssyka Araújo Noronha, and Gisele Karla Sena Guimarães** performed the experiments; **Juliana de Souza Fernandes and Jéssyka Araújo Noronha** carried out the laboratory analyses; **Francisco Denilson Rodrigues Gomes, Juliana de Souza Fernandes, and Jéssyka Araújo Noronha** operated the histological processing; **Bruna Farias Brito** performed all statistical analyses; **Lúcia Daniel Machado da Silva** was responsible for project supervision and coordination; **Juliana de Souza Fernandes and Lúcia Daniel Machado da Silva** wrote the manuscript; **Herlon Victor Rodrigues Silva, Leda Maria Costa Pereira Bersano, and Lúcia Daniel Machado da Silva** were responsible for the final review of the manuscript.

## Conflicts of Interest

The authors declare no conflicts of interest.

## Data Availability

The data that support the findings of this study are available from the corresponding author upon reasonable request.
